# The impact of environmental pollution and climate change on hypertension: a position paper by the European Society of Hypertension (ESH) Working Group on Environment in Hypertension

**DOI:** 10.1093/cvr/cvag061

**Published:** 2026-03-20

**Authors:** Marek Rajzer, Wiktoria Wojciechowska, Andrzej Januszewicz, Yu-Ling Yu, Ji-Guang Wang, Omar Hahad, Andreas Daiber, Manuel Spitschan, Philippe van de Borne, Bojan Jelaković, Ana Jelaković, De-Wei An, Xiao-Fei Ye, Grzegorz K Jakubiak, Michel Burnier, Alexandre Persu, Thomas Weber, Tomasz J Guzik, Reinhold Kreutz, Thomas Münzel, Jan A Staessen

**Affiliations:** First Department of Cardiology, Interventional Electrocardiology and Hypertension, Jagiellonian University Medical College, Krakow, Poland; First Department of Cardiology, Interventional Electrocardiology and Hypertension, Jagiellonian University Medical College, Krakow, Poland; Department of Hypertension, National Institute of Cardiology, Warszawa, Poland; Non-Profit Research Association Alliance for the Promotion of Preventive Medicine, Mechelen, Belgium; Department of Medical Epidemiology and Biostatistics, Karolinska Institutet, Stockholm, Sweden; Research Unit Environment and Health, KU Leuven Department of Public Health and Primary Care, University of Leuven, Leuven, Belgium; The Shanghai Institute of Hypertension, Ruijin Hospital, Shanghai Jiao Tong University School of Medicine, Shanghai, China; Department of Cardiology—Cardiology I, University Medical Center of the Johannes Gutenberg-University Mainz, Mainz, Germany; German Center for Cardiovascular Research (DZHK), Partner Site Rhine-Main, Mainz, Germany; German Center for Cardiovascular Research (DZHK), Partner Site Rhine-Main, Mainz, Germany; Department Health and Sport Sciences, Technical University of Munich, TUM School of Medicine and Health, Chronobiology & Health, Munich, Germany; Max Planck Research Group Translational Sensory & Circadian Neuroscience, Max Planck Institute for Biological Cybernetics, Tübingen, Germany; TUM Institute for Advanced Study (TUM-IAS), Technical University of Munich, Garching, Germany; Department of Cardiology ULB-HUB Erasme, Brussels, Belgium; Department of Nephrology, Hypertension, Dialysis and Transplantation, University Hospital Center Zagreb, Zagreb, Croatia; School of Medicine, University of Zagreb, Zagreb, Croatia; Department of Nephrology, Hypertension, Dialysis and Transplantation, University Hospital Center Zagreb, Zagreb, Croatia; Department of Social Medicine and Epidemiology, Faculty of Medicine, University of Rijeka, Rijeka, Croatia; Non-Profit Research Association Alliance for the Promotion of Preventive Medicine, Mechelen, Belgium; Department of Cardiovascular Medicine, Shanghai Institute of Hypertension, Shanghai Key Laboratory of Hypertension, National Research Centre for Translational Medicine, State Key Laboratory of Medical Genomics, Ruijin Hospital, Shanghai Jiatong University School of Medicine, Shanghai, China; The Shanghai Institute of Hypertension, Ruijin Hospital, Shanghai Jiao Tong University School of Medicine, Shanghai, China; Department of Pharmacology, Faculty of Medical Sciences in Zabrze, Medical University of Silesia, Zabrze, Poland; Faculty of Biology and Medicine, University of Lausanne, Lausanne, Switzerland; Department of Cardiology, Cliniques Universitaires Saint-Luc, Université Catholique de Louvain, Brussels, Belgium; Pole of Cardiovascular Research, Institut de Recherche Expérimentale et Clinique, Université Catholique de Louvain, Brussels, Belgium; Cardiology Department, Klinikum Wels-Grieskirchen, Wels, Austria; Centre for Cardiovascular Science, University of Edinburgh, Edinburgh, UK; Department of Internal and Agricultural Medicine, Jagiellonian University Medical College, Krakow, Poland; Institute of Clinical Pharmacology and Toxicology, Charité-Universitätsmedizin Berlin, Corporate Member of Freie Universität Berlin and Humboldt-Universität zu Berlin, Berlin, Germany; Department of Cardiology—Cardiology I, University Medical Center of the Johannes Gutenberg-University Mainz, Mainz, Germany; German Center for Cardiovascular Research (DZHK), Partner Site Rhine-Main, Mainz, Germany; First Department of Cardiology, Interventional Electrocardiology and Hypertension, Jagiellonian University Medical College, Krakow, Poland; Non-Profit Research Association Alliance for the Promotion of Preventive Medicine, Mechelen, Belgium; Department of Cardiovascular Medicine, Shanghai Institute of Hypertension, Shanghai Key Laboratory of Hypertension, National Research Centre for Translational Medicine, State Key Laboratory of Medical Genomics, Ruijin Hospital, Shanghai Jiatong University School of Medicine, Shanghai, China; Biomedical Science Group, Faculty of Medicine, University of Leuven, Leuven, Belgium

**Keywords:** Hypertension, Environmental pollution, Air pollution, Noise pollution, Light pollution, Climate change, Mitigation strategies, Position paper

## Abstract

Environmental pollution—including air, noise, and light—and progressive climate change are major contributors to global health burdens, responsible for over 9 million premature deaths annuallysa. Among environmental exposures, air and noise pollution show the strongest epidemiological links to hypertension and cardiovascular disease, while emerging evidence also implicates light pollution, toxic metal exposure, and climate-related factors. Hypertension, the leading global cause of mortality, is increasingly recognized as a sentinel marker of environmental damage. Fine particulate matter (PM_2.5_) and road traffic noise exposure are associated with significant increase in hypertension prevalence and incidence. While historical guidelines overlooked environmental contributors, recent updates by the European Society of Hypertension (ESH) and European Society of Cardiology (ESC) have integrated environmental risk factors into hypertension management frameworks.

This position paper from the ESH Working Group on Environment and Hypertension synthesizes current evidence on the epidemiology and pathophysiology of environmental pollution in the development of hypertension. It highlights the mechanistic pathways involving oxidative stress, vascular dysfunction, and neurohormonal dysregulation triggered by pollution exposure. Importantly, the paper outlines mitigation strategies at both population and individual levels, including legislative initiatives, urban planning, and personal exposure reduction techniques.

Considering hypertension as an early manifestation of environmental harm offers a critical opportunity for preventive intervention. It is vital to emphasize strict blood pressure control, enhanced screening in high-risk populations and the integration of environmental exposure monitoring into clinical practice. This comprehensive document seeks to raise awareness among healthcare professionals and inform evidence-based strategies for reducing pollution-related hypertension and cardiovascular morbidity.

## 1. Introduction

Human activities have led to increased environmental pollution, including air, noise, light, water, and soil pollution, as well as progressive climate change.^1^ Substantial number of people are exposed to these phenomena that are, according to a WHO report, responsible for more than 12.6 million premature deaths in 2012.^2^ The Global Burden of Disease study confirms magnitude of the problem, estimating the number of deaths attributable to environmental pollution risk factors at 9 million in 2015^3,4^ The strongest epidemiological evidence linking environmental pollution to the incidence and prevalence of hypertension and coronary artery disease relates to air and noise pollution; however, negative effects have also been confirmed for light pollution, climate change, and soil and water pollution^5–9^ Besides the epidemiological data linking selected environmental pollution risk factors with hypertension, there is also convincing pathophysiological evidence for common mechanisms that increase blood pressure (BP) for air, noise, and light pollution as well as climate change and toxic metals. All these factors are currently encompassed within the mosaic theory of hypertension.^5^

Based on the Lancet Commission on the Global Burden of Disease (GBD), hypertension is the leading cause of death^10^ as well as leading risk factor of established cardiovascular diseases (CVD) like myocardial infarction^11^ and stroke.^12^ Based on epidemiological studies hypertension might be also recognized as an early manifestation of environmental harm. Strong epidemiological evidence shows that for every 10 µg/m^3^ in fine particulate matter (PM_2.5_) concentration, the prevalence of hypertension rises by 4%. In the two highest quartiles of PM_2.5_ exposure (25.4–97.0 μg/m^3^), the risk of hypertension exceeded 30% compared to the lowest exposure levels (2.6–14.0 μg/m^3^).^13^ Each interquartile range (IQR = 16.80 μg/m^3^) increase in the 1-year average PM_2.5_ concentration, is associated with a 17% increase in hypertension incidence. The total population-attributable fraction (PAF) of long-term PM_2.5_ exposure for hypertension incidence is 23%.^14^

In 2014, the European Environment Agency estimated that over 900 000 new cases of hypertension per year could be due to environmental noise exposure.^15^ According to WHO report, every 10 dB increase in environmental noise raises the prevalence of hypertension by 5%.^16^ A recent study found that long-term exposure to road traffic noise was associated with an increased risk of primary hypertension, an effect that was further amplified when combined with air pollution, leading to a 22% increase in hypertension incidence.^17^

In a European Commission document dated 15.05.2021, it is stated that under European Union law, the Green Deal ambitions, and in synergy with other initiatives, the EU aims to reduce the health impacts of air pollution on premature deaths by more than 55% and the proportion of people chronically disturbed by transport noise by 30% until 2030.^18^ This recommendation has led to further regulations, including guidelines on how member states should report the health benefits of noise pollution reduction.^19^

Initially, the role of environmental pollution in hypertension and CVD was not recognized in medical guidelines, as the traditional model of CVD aetiology focused on lifestyle-related or genetic causes. Indeed, the lifestyle-related and environmental factors that contribute to the development of various diseases form part of a larger entity known as the exposome, which encompasses all non-genetic factors. It was only in 2015 that the expert position paper of the European Society of Cardiology (ESC) emphasized the impact of the environment on cardiovascular disease (CVD), focusing solely on air pollution.^20^ The 2021 ESC Cardiovascular Disease Prevention Guidelines were the first recommended in cardiovascular prevention to avoid long-term stays in regions with high levels of air pollution especially for high-risk patients. At the population level, guidelines advise screening programs to detect CVDs in regions with high pollution levels.^21^ The ESH 2023 Guidelines have underscored the importance of environmental factors in hypertension, placing them on par with genetic and lifestyle factors.^22^ Similarly, 2024 ESC Guidelines for the management of elevated BP and hypertension further highlighted the role of environmental pollution in elevated BP and hypertension.^23^ The role of environmental pollution in hypertension is presented comprehensively and practically in International Society of Hypertension position paper on lifestyle management endorsed by the World Hypertension League and the European Society of Hypertension.^24^

To date, there is no publication that addresses a broad spectrum of environmental pollutants while focusing on their role in hypertension. In a previously published ESC document, the impact of air pollution,^20^ noise pollution,^25^ and heat extremes^26^ were addressed with regard to general aspects of CVD. Accordingly, this position paper by the ESH Working Group on Environment in Hypertension summarizes the current state of knowledge on environmental factors contributing to hypertension. It presents evidence that environmental exposures are not merely ancillary risks but may represent core, causal drivers of the global hypertension pandemic. The document also offers practical recommendations to mitigate these effects at both individual and population levels. To facilitate this, the position paper is divided into five chapters according to the type of exposure.

## 2. Air pollution

### 2.1 Epidemiological evidence

Air pollution had the fourth position as a cause of death globally^10^ (*Table 1*). Systolic blood pressure (SBP) contribution to mortality is increasing in high-, middle-, and lower-income countries, but air pollution role is substantial only in middle- and lower-income countries.^10^ Particulate matter pollution ranks first for total disability-adjusted life years (DALYs) (8.0%) followed by high SBP (7.8%).^31^ Globally, air pollution exposure may lead to an excess mortality rate of 8.79 million per year, of which ∼790.000 are in Europe, with 48% due to CVDs.^32–34^ The WHO estimates that tobacco smoking causes an excess death rate of 7.2 million per year, making air pollution an even larger risk factor.^35^

**Table 1:** Key epidemiological points of air pollution health effects with special focus on hypertension

Global trends	Urban vs. rural populations	Vulnerable populations	Contribution to hypertension prevalence
WHO estimation: 4.2 million premature deaths annually linked to outdoor air pollution (CVD and hypertension—major contributors).^27^ In EU: of 269 000 deaths attributable to PM2.5 almost 143 000 are due to ISH or stroke.^28^	Urban populations at higher risk due to greater exposure to pollutants. Significantly higher BP levels in residents of areas with high traffic and industrial activities.^29^	Children, elderly, men and individuals with heart and lung diseases are particularly susceptible to the hypertensive effects of air pollution.^30^	Air pollution is estimated to account for approximately 10–15% of hypertension cases globally.^10^ The association is dose-dependent, with higher exposure correlating with greater increases in BP.

WHO, World Health Organization; CVD, cardiovascular diseases; BP, blood pressure.

The main components of air pollution are listed in *Table 2*. There is a growing epidemiological evidence on relation of air pollution and hypertension, with differences in the effects of short- and long-term exposure. Most evidence of the impact of air pollution on BP or hypertension relates to PM_2.5_ particles. Long-term exposure to PM_2.5_ was significantly associated with hypertension, while other air pollutants did not.^36^ However, long- and short-term exposure to PM_10_, PM_2.5_, and NO_2_ was positively associated with diastolic blood pressure (DBP).^36^ There is also evidence from a large cross-sectional study that long-term exposure to ozone (O_3_) can increase the prevalence of hypertension and BP levels.^37^ However, the data on the relationship between ozone and BP are inconsistent.^38^ Long-term exposure to high level of sulphur dioxide (SO_2_) increased the risk of hypertension incidence^39^ and BP increase.^40^ In a Chinese Health Study, prehypertension was more strongly related to long-term ambient air pollution [PM10, SO_2_, nitrogen dioxides (NO_2_), and O_3_] exposure than hypertension, which may be due to their non-linear relationship. Associations of SBP with air pollution were stronger in women and older participants, who are more vulnerable to environmental pollutants.^41^ One-year exposures to PM_10_, PM_2.5–10_, PM_2.5_ absorbance, and NOx estimated by land-use regression models were associated with higher diastolic BP in elderly residents of Taipei.^42^ Observed short-term effects of air pollution (PM_10_, PM_2.5_, SO_2_, NO_2_, CO, O_3_) are more pronounced in young individuals, while long-term in the elderly.^36,41,43^ Each IQR μg/m^3^ increase in PM_2.5_ exposure was also related to increases of SBP by 2.54 mmHg [95% confidence interval (CI): 1.99, 3.10], and DBP by 1.36 mmHg (95% CI 1.04, 1.68). Additionally, per each IQR μg/m^3^ increase in the chemical components of PM2.5 mass [sulphate (SO_4_^2−^), nitrate (NO_3_^−^), ammonium (NH_4_^+^), organic matter (OM), black carbon (BC)] there were associated increments of SBP (1.39–3.87 mmHg) and DBP (0.83–2.11 mmHg)^14^ (more data are available in Supplementary material online, *Table S1*).

**Table 2:** Components of air pollution

Gaseous pollutants	Particular matter	Other
Nitrogen dioxide (NO_2_)	PM10	Lead (Pb)
Sulphur dioxide (SO_2_)	PM 2.5	
Carbon monoxide (CO)	PM 0.1	
Ozone (O_3_)		

Ozone (O_3_) is to a large extent a secondary pollutant, and its concentration largely depends on sun (UV) exposure of various compounds.

There is a nonlinear association of BP with air pollution due to differences in susceptibility across age groups. Differences may be also due to study design, population, and environment. Air pollution's effect on older people is weakened by higher cardiovascular risk and comorbidities. Despite these important confounders, a longitudinal study of elderly Chinese people found strong evidence of an increase in systolic BP due to air pollution, particularly PM_2.5_.^44^ Younger adults are more susceptible to hypertension incidents, while those with established cardiovascular risk, hypertensives, and the elderly are more vulnerable to BP rise and acute events due to air pollution. The long-term effect of air pollutants is difficult to observe due to the mixed impact of other risk factors like traffic noise, genetic susceptibility, dietary intake, and outdoor behaviours. In summary, epidemiological studies consistently show a relation between air pollution exposure and increased hypertension prevalence.^36,41,43,44^ This is in contrast to the ESCAPE study, which showed only a weak influence of PM_2.5_ exposure on self-reported hypertension prevalence but not confirmed by BP measurement.^45^ Other studies indicated that air pollution may contribute to the development of hypertension during pregnancy^46^ and negatively influence birth weight.^47^

### 2.2 Pathophysiology

Air pollution is linked to hypertension through various mechanisms. Gaseous pollutants and small PM_2.5_ enter the alveolar-capillary membrane, leading to endothelial dysfunction, inflammation, and increased BP,^48,49^ even in healthy individuals.^50^ This also results in acute autonomic nervous system (ANS) imbalance, which increases heart rate and decreases heart rate variability. BP increase in response to exposure is mediated by ANS and its imbalance with domination of sympathetic over parasympathetic tone. Autonomic innervation of airways supports this explanation. DBP rise is sustained as it persists during 5-h after car gas exhaustion inhalation, and is mitigated by α-adrenergic receptor antagonism.^50^ PM_2.5_ exposure may cause an acute increase in BP due to oxidative stress-mediated impairment in nitric oxide bioavailability, resulting in vasoconstriction. However, the BP change in exposure studies was unrelated to inflammatory biomarkers and not attenuate by Vitamin C pre-treatment.^48,50–54^ Ozone exposure is also a possible cause of oxidative stress, leading to short-term increases in cardiovascular mortality, especially when accompanied by PM_2.5_ increase.^55,56^ Short-term O_3_^-^ exposure leads to a rise in BP in healthy adults.^57^ Elevated BP and O_3_-exposure may be linked by increased serotonin-induced vasoconstriction and decreased acetylcholine-induced vasodilation.^58^

In a cross-over study, a 2 h walk along a commercial street in London was associated with detrimental effects on pulsatile haemodynamics (pulse wave velocity, augmentation index), in contrast to a 2 h walk in Hyde Park.^59^

**Figure 1:**
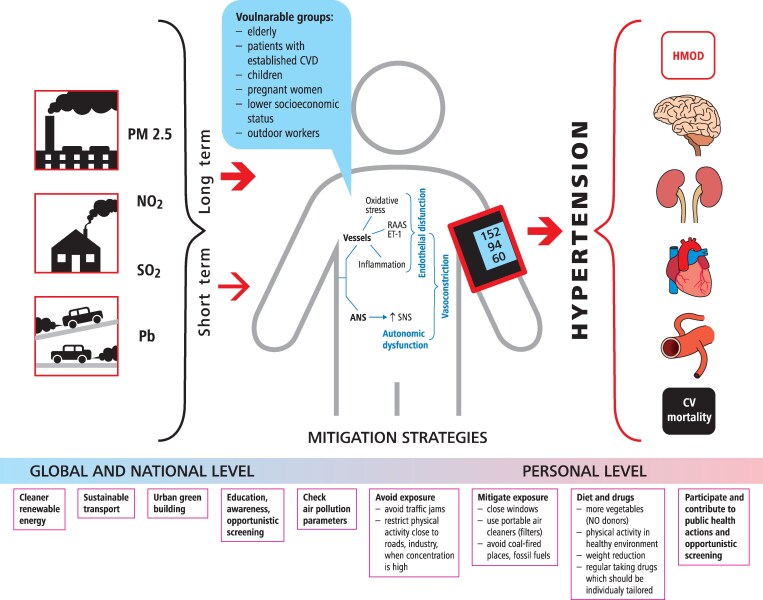
Air pollution and hypertension. ANS, autonomic nervous system; SNS, sympathetic nervous system; RAAS, renin–angiotensin–aldosterone system; ET, endothelium.

Air pollution activates immune cells to release pro-inflammatory cytokines (IL-6, TNF-α, IL-17, IFN-γ)^60–62^ and activates immune cells, which contributes to systemic inflammation^63^ and leads to vascular dysfunction and calcification. These responses drive vascular damage, calcification,^64^ sympathetic nervous system (SNS) activation, and pro-oxidative mediator release, directly raising BP and promoting hypertension.^61^ It has been estimated that 15–27% of cardiovascular effects may be linked to immune mechanisms.^65^ In otherwise healthy young individuals (aged 15–21), long-term exposure to higher air pollution levels related to inhabitance in a highly air polluted city (Krakow) was associated with elevated CRP, hs-CRP, fibrinogen, and homocysteine, as well as higher systolic and pulse pressure and lower heart rate in males, highlighting early immune-driven cardiovascular changes with sex-specific responses.^66^ In particular, accumulation as well as indirect proinflammatory effects of PM_2.5_ and PM_10_ on immune cells accumulate over time leading to accelerated immunosenescence,^63^ which contributes to hypertension and CVD. Therefore, ageing of immune and cardiovascular systems needs particular therapeutic attention in subjects exposed to air pollution.^67^ The potential complex mechanisms by which air pollution induces hypertension are summarized in *Figure 1*.

### 2.3 Mitigation strategies

The 2021 ESC guidelines on cardiovascular disease prevention in clinical practice recommend avoiding long-term exposure to regions with high air pollution for patients at high risk for CVD (class IIb, level of evidence C).^21^ They also suggest implementing CVD risk screening programmes in regions with high pollution levels (IIb, C). Policy interventions at the population level include reducing PM and gaseous pollutants as well as carbon dioxide emissions, restriction of fossil fuel use to reduce CVD mortality and morbidity (I, C). Air pollution is also a significant environmental risk factor for hypertension, as mentioned in recent ESH and ESC guidelines.^22,23^ The recommendations from cited above guidelines have a level of evidence ‘C’ which means are based on experts’ opinion supported by data from small studies, retrospective studies, and registries. These recommendations aim to reduce air pollution and improve healthcare interventions as summarized in *Tables 3–4* and *Figure 1*.

**Table 3:** Mitigation strategies for air pollution

International guidelines	National, local and societal measures	Personal instructions
WHO global air quality guidelines. Reduce exposure to key pollutants like PM2.5 and NO_2_.^68^ European Union Directive on ambient air quality and cleaner air for Europe.^69^ European Environmental Agency (EEA) supports EU member states in achieving compliance with air quality standards.^70^	Regulatory frameworks Countries implement air quality regulations, such as the Clean Air Act in the U.S.^71^ Local government’s regulations diminishing the amount of exposure by traffic limitations and urban landscape reorganizations. Cities are increasingly adopting green infrastructure, promoting public transportation, and implementing low-emission zones to reduce overall pollution levels. Community Initiatives to monitor air quality and advocate for policies that protect vulnerable populations.	The use of home air purifiers, which provide reasonable protection from both indoor and outdoor pollution, was associated with a reduction in SBP.^72^ Checking PM and gaseous air concentrations before planning extended out-door activity as part of our new routine.^73^ Using removable energy. Saving energy at home. Reducing traffic transportation. Avoiding physical activity close to heavy traffic roads. Collaborating and participating in public health actions.^21^ Wearing N95 facial masks does not provide protection from acute effect of air pollutants on CV system.^74^

**Table 4:** Healthcare interventions

Medical staff interventions	Community health initiatives	Short summary and recommendations for healthcare providers
Screening and monitoring BP monitoring and screening for individuals living in high-pollution areas for early detection and adequate management of hypertension. Patient education Educate patients about the risks of air pollution and provide strategies for reducing exposure. Pharmacological interventions Optimalization of antihypertensive treatment to obtain BP goal. Lifestyle counselling Encouraging physical activity, healthy diet, and stress management.	Collaborative programs partnerships between health and environmental agencies to promote awareness and prevention of pollution-related health risks. Research and data sharing collaborative research efforts for better understanding of the relationship between air pollution and hypertension.	Assess environmental risk Incorporate individual environmental exposure assessments into routine health evaluations, especially in high-risk patients. Educate patients Inform patients about the health effects of air pollution and effective strategies to reduce exposure. Recommend reducing time spend in high air pollution areas in patients with hypertension and CVD. Collaborate with public health officials Work with public health authorities to advocate for policies that improve air quality and protect public health.

BP, blood pressure; CVD, cardiovascular disease.

All major components of air pollution may contribute to increases in BP and the development of hypertension; moreover, they typically occur as a mixture. Interventional studies demonstrating BP reductions with decreased exposure are scarce, with the strongest evidence available for reductions in PM_2.5_.^75^

In addition to the necessity for societal reforms, individual-level strategies can provide significant protection, particularly for those living in high-exposure environments. Scientific Statement from the American Heart Association^76^ recommends several evidence-based interventions, including those supported by data from randomized double-blind studies. These include the use of high-efficiency particulate air (HEPA) filters indoors, capable of reducing indoor PM_2.5_ concentrations by up to 60%. Wearing N95 masks during periods of elevated outdoor pollution or while commuting can substantially lower personal exposure to particulate matter. Limiting or avoiding outdoor activities during peak pollution hours, especially in areas with heavy traffic, is also advisable. The modifying effect of physical activity on the relationship between air pollution and hypertension remains inconsistent. Some studies suggest that physical activity does not alter the adverse impact of air pollution on hypertension,^77^ whereas others indicate that increased physical activity may attenuate the negative effects of air pollution on BP and hypertension risk.^78,79^ Under low PM_2.5_ exposure, subjects with highest level of physical activity had significant decrease in systolic BP, while physical activity at high PM_2.5_ concentration was associated with an increase in SBP.^80^ International Society of Hypertension position paper recommended exercise in parks and gardens away from busy roadways and limit time spent outdoors during highly polluted periods.^24^

Furthermore, dietary interventions enriched with natural antioxidants and omega-3 fatty acids may counteract pollutant-induced oxidative stress and inflammation lowering BP.^81^ The DASH diet, as well as the Mediterranean diet, may reduce the risk of air pollution-related hypertension.^82,83^

Importantly, rigorous management of cardiovascular risk factors—such as hypertension, diabetes, and dyslipidaemia—can strengthen individual resilience against the adverse effects of air pollution.^84^

While these personal measures offer important protection, they cannot replace the need for regulatory and structural mitigation efforts.^24^ It is important to emphasize that a comprehensive strategy is essential. Urgent societal reforms aimed at reducing fossil fuel use and achieving the World Health Organization’s air quality standards^68^ must go hand in hand with personal-level interventions. This dual approach is critical for reducing the cardiovascular burden of air pollution, particularly among vulnerable and susceptible populations (*Table 3*).

## 3. Noise pollution

### 3.1 Epidemiological evidence

A large body of evidence confirms noise as a major public health risk. According to WHO traffic noise causes up to 1.6 million healthy life years lost annually.^85^ Noise-related social costs are estimated at 1 trillion EUR annually.^86^ The European Environmental Agency (EEA) reports 53 million adults affected by noise, 34 million with sleep disturbances, and 1.7 million new cases of hypertension yearly, resulting in 80 000 hospitalizations and 18 000 premature deaths. According to a report from the year 2014, 270 million Europeans were exposed to nighttime noise levels (*L*_night_) exceeding 40 dB.^87^ These numbers have been updated by the WHO Noise Guidelines for the European Region in 2018: more than 100 million people in Europe are exposed to *L*_den_ levels above 55 dB; for nighttime road traffic noise, over 72 million Europeans are exposed to *L*_night_ levels above 50 dB.^88^

According to EEA report 113 million EU citizens are affected by high, i.e. over 55 dB *L*_den_ (level during day evening and night) road traffic noise levels. The number of affected by high levels of railway noise exceeded 22 million, while 4 million are exposed to high levels of aircraft noise.^89^ In Europe, long-term environmental noise exposure might cause 48 000 new cases of ischaemic heart disease per year, high annoyance in 22 million people and chronic sleep disturbance in 6.5 million. Even if studies applied varied methodology, the evidence consensually indicates that environmental noise, particularly from traffic, enhances the risk of hypertension.^90,91^

A WHO expert group's meta-analysis found a relative risk (RR) of 1.05 (95% CI 1.02–1.08) per 10 dB *L*_den_ of road traffic noise for prevalent hypertension, indicating a significant but modest effect.^16^ This association has been reinforced by several studies, including Fu et al.'s finding that both community and occupational noise were linked to hypertension with an odds ratio (OR) of 1.06 (95% CI 1.04–1.08).^92^ Aircraft noise has also been identified as a significant contributor to hypertension, with a higher hazard ratio (HR) of 1.36 (95% CI 1.02–1.82) for hypertension and increased BP, namely 10 dB(A) increase in the day–evening-night noise level (*L*_den_) was associated with a 1.93 mm Hg increase in SBP (95% CI 0.79–3.08) and a 1.08 mm Hg increase in DBP (95% CI 0.27–1.88).^93^ Research from seven European countries showed an RR of 1.03 (95% CI 1.01–1.06) per 10 dB(A), *L*_night_ for hypertension concerning aircraft noise.^94^ However, the relationship between noise and hypertension is not uniformly linear, with some populations showing a more nuanced association. Shin et al. reported a small but significant increase in the risk of incident hypertension due to road traffic noise (HR 1.02, 95% CI 1.01–1.03),^95^ while a Danish cohort study found no significant relationship (HR 0.999, 95% CI 0.980–1.019).^96^ Two US-based cohort studies found that hypertension incidence increased with a noise exposure level (HRs of 1.04 and 1.03).^97^ A study of a US cohort of Mexican Americans found a HR of 1.10 for 24-h noise exposure per 11.6 dB increase.^98^ Variations in findings may be due to differences in population characteristics, exposure and outcome measurement techniques, or concurrent exposure to other environmental risk factors. A UK Biobank study showed that increased road traffic noise levels were associated with modest increases in SBP (0.77%) and DBP (0.49%), for every 10 dB increase of road traffic noise an approximate increase of 1.06 in SBP and 0.40 mmHg in DBP was observed.^84^ Moreover, combined effect of road traffic noise exposure and air pollution on increased hypertension incidence was documented.^17^ A pooled analysis of nearly 45 000 individuals indicated that increased noise exposure could lead to higher BP,^99^ also supported by studies showing higher BP from indoor nocturnal noise, at 10 dB(A) increase of nocturnal noise exposure SBP and DBP increased, respectively, by 3.03 mmHg (95% CI 1.14–4.92, *P* = 0.002), and 0.68 mmHg (95% CI − 0.65 to 2.01, *P* = 0.317)^100,101^ (more data are available in Supplementary material online, *Table S1*).

Temporary reductions in aircraft noise, such as during the COVID-19 pandemic, have been shown to mitigate the adverse effects of noise on BP, suggesting that even short-term improvements in noise levels could have measurable health benefits.^102^ A meta-analysis found an RR of 1.13 for hypertension in response to noise, but moderate heterogeneity across studies was noted.^103^ Dzhambov et al. observed suggestive increases in systolic and diastolic BP among children exposed to road traffic noise at school or home. Still, the high heterogeneity among studies and evidence of publication bias complicate the interpretation of these findings.^104^ A meta-analysis highlighted a 7% increased risk of hypertension associated with road traffic noise exposure but found no significant link with SBP or DBP in both adults and children.^105^ The consensus is that environmental noise exposure, particularly from road traffic and aircraft, is a risk factor for hypertension (*Figure 1*), with further research needed to understand the dose-response relationship and factors contributing to the heterogeneity of study outcomes.^106–109^ According to the WHO document, there is a relationship between environmental noise and adverse birth outcomes, including low birth weight, small gestational age, preterm birth and congenital malformations.^110^ Some of them are risk factors for hypertension in adulthood. Moreover, environmental noise may be a novel risk factor for early onset of severe preeclampsia.^111^

### 3.2 Pathophysiology

**Figure 2:**
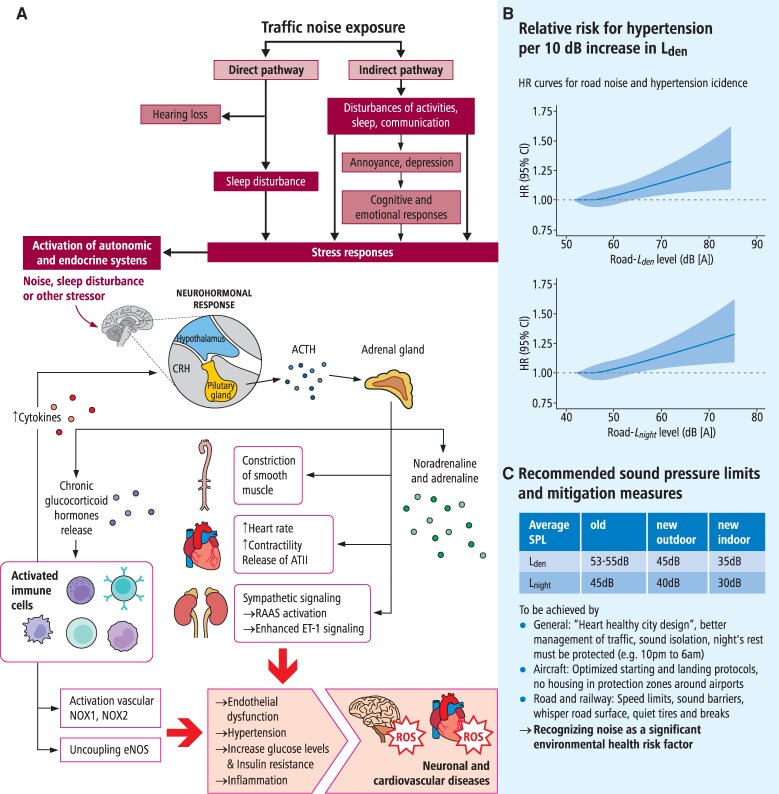
Noise pollution and hypertension. (*A*) Noise induces physiological responses through two distinct pathways (direct and indirect), which converge via the activation of stress responses. Chronic activation of these stress response pathways can lead to the emergence or exacerbation of cardiovascular risk factors, ultimately contributing to cardiovascular disease. In response to stress, CRH is released in the brain. Upon uptake of CRH by the pituitary gland, ACTH is secreted, stimulating the adrenal pituitary gland to release neurotransmitters and glucocorticoids. These substances can directly affect blood pressure and activate immune cells, which in turn release cytokines that feedback to the brain. CRH, corticotrophin-releasing hormone; ACTH, adrenocorticotropic hormone; RAAS, renin–angiotensin–aldosterone; ET-1, endothelin-1. This scheme is summarized from references.^113–116^ Reused from reference^117^ with permission. (*B*) Relative risk of hypertension due to traffic noise, based on meta-analyses from an Umbrella + review (2023) estimating the association between road traffic noise and cardiovascular disease.^118^ The exposure-response curves illustrate the association between road traffic noise and incident primary hypertension (based on the fully adjusted model). Left: Weighted average 24-hour road traffic noise level (*L*_den_); Right: Average nighttime road traffic noise level from 23:00 to 7:00 (*L*_night_). *L*_den_ = weighted average 24-h road traffic noise level; *L*_night_ = average nighttime road traffic noise level from 23:00 to 7:00. Reused from reference^17^ with permission. (*C*) WHO and EEA recommendations for traffic noise limits and mitigation measures suggested by the guideline development group (see main text for references).

The mechanism of noise-induced adverse health effects is best described by the noise stress response concept proposed by Babisch in 2003^112^ (*Figure 2*). According to this concept, the direct noise pathway is operative upon intense sound pressure exposure (above 90 dB) leading to inner ear (cochlear) damage and hearing loss. In comparison, the indirect noise pathway is triggered by lower sound pressure levels (below 80 dB) and characterized by systemic health effects such as annoyance and sleep disruption. A key mechanism of the indirect pathway is the release of stress hormones by pituitary-adrenal-cortical axis (cortisol) and sympathetic-adrenal-medullary axis (catecholamines)^119^ activation. These stress hormones were also found in noise-exposed humans (for cortisol^120,121^ and catecholamines^122,123^) and animals, where increased levels of corticosterone, adrenaline, and noradrenaline were established.^124,125^ A recent study identified a mechanistic link between transportation noise exposure and activation of the stress response centre in the brain, the amygdala.^126^ The authors demonstrated that higher aircraft and road noise (>*L*_den_ 55 dB) increased amygdala activity, that was associated with vascular inflammation and subsequently within the next 5 years with more frequent major adverse cardiovascular events (MACE) (HR & 1.34, 95% CI 1.15–1.57, per 5 dB).^126^

Noise contributes to the development of hypertension by the pathological mechanisms summarized in *Figure 2A*.^113,127^ Adrenaline and noradrenaline activate cardiac β1-receptors and vascular α1-receptors, leading to an increase in BP. Activation of glucocorticoid receptors also increases vessel resistance and increases BP. Catecholamines activate the renin–angiotensin–aldosterone system (RAAS) producing the potent vasoconstrictor angiotensin-II, a strong inducer of hypertension. Catecholamines and angiotensin-II, via activation of their α1- and AT1-receptors, trigger G-protein coupled activation of phospholipase C that catalyses the conversion of phosphatidylinositol-4,5-bisphosphate to inositol 1,4,5-trisphosphate and diacylglycerol. The latter is one of the most potent endogenous activators of protein kinase C, the key enzyme for phagocytic NADPH oxidase (NOX-2) activation via phosphorylation of the regulatory subunit p47phox. NADPH oxidase-derived superoxide anion radicals react with nitric oxide, release by the endothelial nitric oxide synthase, yielding peroxynitrite.^128^ Peroxynitrite together with hydrogen peroxide from monoamine oxidases (using catecholamines as substrates) inhibits the activity of the prostacyclin synthase via tyrosine nitration of the enzyme^129^ and causes oxidative impairment of the nitric oxide-cGMP vasodilatory system by uncoupling of endothelial nitric oxide synthase (eNOS) and oxidative inactivation of the soluble guanylyl cyclase leading to increased vascular tone, vascular resistance and accordingly to higher BP.^130^ Moreover, peroxynitrite and hydrogen peroxide mediate adverse phosphorylation of eNOS at Thr495 and Tyr657 residues by redox-activation of protein kinase C and protein tyrosine kinase 2.^131^ Oxidative stress conditions also increase gene expression levels of endothelin-1, another potent vasoconstrictor that also generates diacylglycerol.

The above-described processes promote vascular inflammation, also by cortisol/corticosterone resistance and direct effects of catecholamines on immune cells, as suggested by higher levels of circulating pro-inflammatory cytokines interleukin-6 and -1β, as well as expression levels of NFkB in noise-exposed mice^124,125,132^ and higher C-reactive protein in humans.^133^ Through these inflammatory mechanisms aircraft noise exposure lead to pro-inflammatory vascular transition, escalate vascular dysfunction, and promote negative cardiac remodelling after myocardial infarction.^134^ Impaired sleep and dysregulated circadian rhythms may further aggravate the pro-inflammatory and oxidative stress conditions by phase and amplitude shifts of genes encoding for antioxidant or reactive oxygen species producing enzymes and pro-/anti-inflammatory genes.^135^ All these processes together significantly contribute to the development of hypertension and atherosclerosis by noise exposure, also explaining the additive damage of noise in mice with pre-established arterial hypertension.^136^

### 3.3 Mitigation strategies

All mitigation measures are based on observational and interventional studies that provide the basis of the WHO/EEA recommendation. The current WHO Guidelines on Environmental Noise for the European Region have formulated concrete recommendations for protecting human health from environmental noise from various sources (*Figure 2*).^88^ The more recent EEA report and recommendation for environmental noise in Europe highlights similar mitigation measures.^137^ These recommendations represent expert consensus acknowledging that there is moderate or low quality of evidence from interventional studies. Different types of interventions like type A source interventions (e.g. change in traffic flow rate, track restrictions), type B path intervention (e.g. dwelling insulation, barrier construction), type C changes in infrastructure (e.g. road tunnels), and type D other physical interventions (e.g. availability of quite side to the dwelling) were analysed and summarized in separate tables indicating that they are health effects of such interventions in: reducing noise annoyance, sleep disturbance, and cardiovascular disease.^88^ It is important to mention that the development process of the WHO guidelines followed a strict methodology using the Grading of Recommendations Assessment, Development and Evaluation (GRADE) approach and that the recommended noise levels are also applicable in other regions, because studies from America, Asia, and Australia were considered in addition to evidence from European studies on noise-related health effects.^88,138^ To avoid adverse health effects, the guidelines recommend reduction of average daily:

- road traffic noise <53 dB *L*_den_ and <45 dB *L*_night_,
- railway noise <54 dB *L*_den_ and <44 dB *L*_night_,
- air traffic noise <45 dB *L*_den_ and <40 dB *L*_night_.^88,139^

According to the German Aerospace Centre (DLR), aircraft noise should induce less than one additional wake-up response per night and cause no noticeable waking up (*L*_max_ ≤ 65 dB). A DLR study showed that nocturnal aircraft noise levels of >33 dB increase the probability of waking up compared to the spontaneous probability of waking up.^140^

Concrete measures for reducing aircraft noise-associated health effects are prevent housing in the protection zones around airports (defined by national legislation) and offering cost-free active noise protection measures to residents who live just outside the protection zones. The continuous descent approach, flying higher, and landing steeper or GPS-guided approach over sparsely populated areas would be measures that could be implemented by airport operators. The legally defined nighttime rest period (e.g. from 22:00 to 6:00) should be aimed at shifting air traffic, where unavoidable, more into the daytime (Detailed guidance on the application of the Balanced Approach is provided in the ICAO Doc 9829, Guidance on the Balanced Approach to Aircraft Noise Management). For road and railway noise reduction in general, better noise isolation windows and house façades, as well as sound barriers are recommended measures. Highly effective are speed limits (speed reduction from 50 to 30 km/h) resulted in noise drop by 1.6 dB during day and 1.7 dB at night at the loudest facade point.^141^ Whisper road surface (may resulted in noise reduction by 6.8 dB compared to a conventional road surface),^142^ quiet tires [2 dB(A) total noise reduction] and brakes^143^ represent other effective mitigation measures.^142^ Mitigation measures are summarized in *Figure 2*.

## 4. Light pollution

### 4.1 Epidemiological evidence

#### 4.1.1 Challenges in estimating light exposure

**Figure 3:**
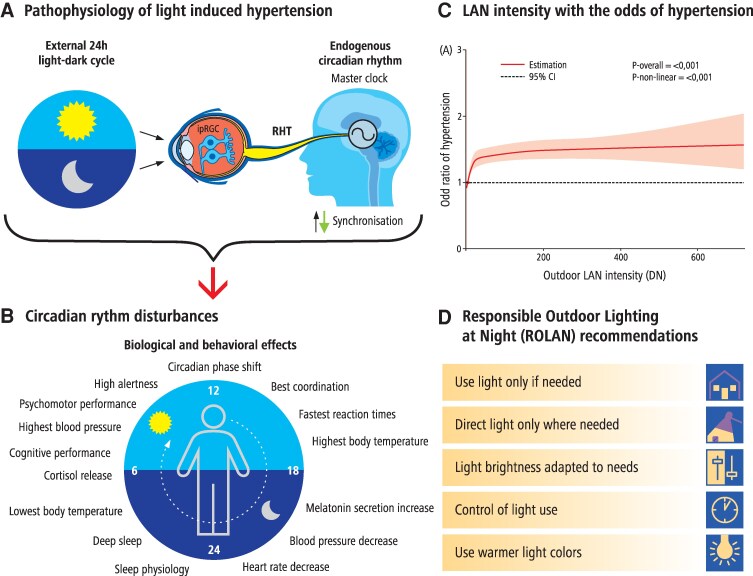
Light pollution and hypertension. (*A*) Pathophysiology of light pollution effects including the central role of the intrinsically photosensitive retinal ganglion cells (ipRGCs) and retinal hypothalamic tract (RHT), (*B*) circadian rhythm disturbances components and consequences, (*C*) light pollution and odds of hypertension according to reference,^148^ and (*D*) light pollution mitigation strategies.

Light exposure can be challenging due to eye, head and trunk movements, pupil size, and other individual factors which change how much light reaches the retina. A similar challenge exists in converting measurements of environmental nighttime light into personal light exposure. Several large-scale studies from the UK Biobank have examined personal light exposure from wrist-worn devices and its consequences on health, including the finding that bright light exposure at night (LAN) and/or dim light exposure during the day can be associated with mental health,^144^ diabetes,^145^ and overall mortality.^146^ Most large-scale studies do not have personal light exposure data available, requiring satellite imagery, which is limited in spatial and temporal resolution (but can be correlated with exposure on Earth under certain circumstances^147^) and may not capture the correct wavelengths relevant for the melanopic impact on physiology (*Figure 3*).

Notably, one field study in a cohort of 522 older adults within the Chicago Healthy Aging Study (CHAS) showed that nighttime light exposure as measured with wrist-worn actigraphy predicts hypertension (OR 1.74; CI 1.21–2.52).^149^

#### 4.1.2 Epidemiological findings with satellite imagery

Several large-scale studies have examined the link between cardiovascular health and nighttime light exposure estimated with satellite imagery. In a study with 58 692 participants in Hong Kong, coronary heart disease (CHD) was found to be related to nighttime outdoor light exposure, where brighter outdoor light exposure increasing the risk of CHD.^150^ A cohort study with 13 507 participants in China within the China Health and Retirement Longitudinal Study (CHARLS) showed that light exposure can increase the odds of hypertension as well as increase in SBP (0.59 mmHg/IQR, 95% CI 0.027,1.157) and DBP (0.85 mmHg/IQR, 95% CI 0.525,1.180) (more data available in Supplementary material online, *Table S1*). Participants with the highest quartile of outdoor LAN intensity had 1.31-fold increased odds of hypertension (95% CI 1.08–1.58) compared to the lowest quartile (*Figure 3*).^148^ Finally, the Catalan GCAT cohort study showed a clear relationship between melanopic outdoor illumination, hypertension, and other cardiometabolic diseases.^151^

### 4.2 Pathophysiology

Light is a key stimulus for human physiology. In addition to enabling us to see, it is a zeitgeber, synchronizing our internal clock with environmental time given by the solar day, and can lead to the melatonin suppression. Light exposure in the evening and at night can have negative consequences, disrupting physiology. In addition to the circadian and neuroendocrine effects of light at the wrong time, light exposure at night can also influence cardiovascular physiology^152^ and health.^153^

#### 4.2.1 From environmental exposure to retinal stimulus

Light in the environment enters the eye through the lens and gets imaged on the retina. Notably, different photoreceptors in the retina have different wavelength preferences: The cones and rods underlie visual processes, mediating our vision of colour, space, and motion. In addition, around 25 years ago, a separate class of photoreceptors was discovered, the intrinsically photosensitive retinal ganglion cells (ipRGCs) expressing the photopigment melanopsin.^154–156^ The ipRGCs are sensitive to light independent of the cones and rods and are sensitive to short-wavelength light.^157,158^ Activation of the ipRGCs mediate various physiological effects, including circadian phase shifting, melatonin suppression, alertness, and sleep (*Figure 3*). These ‘non-visual’ effects are subject to several factors, including pupil size^159,160^ and age.^161^ A recent study found that in melatonin suppression, there are individual differences of up to a factor of 30.^162^

#### 4.2.2 Time-dependent effects of light

The exact effect of light exposure depends on the time of day, or more precisely, an individual's circadian time.^163,164^ Broadly, exposure to light in the morning can boost alertness, reduce sleep inertia, and advance the circadian clock. Light in the evening and at night can delay sleep, delay the timing of the circadian clock, and cause the acute suppression of the hormone melatonin, which is not produced during the day and rises in production roughly one to three hours before habitual bedtime.

#### 4.2.3 Light exposure at night

Various terms exist in the literature that capture the availability of light at the wrong time.^165^ For environmental light exposures due to outdoor lighting at night, the terms ‘light pollution’ and ‘artificial light at night’, abbreviated as ALAN, are used, and often imply effects beyond human health, including on fauna and flora. To understand the impact of light exposure at night on human well-being, the exact nature of the exposure is inconsequential at the level of stimulus, as the retina—beyond the ipRGCs being maximally stimulated by short-wavelength light peaking near 490 nm^157,158^—does not distinguish between photons that enter the bedroom window, photons from hallway illumination bleeding into the bedroom, or photons that are emitted from smartphones used at night. A study conducted among Japanese older people in Nara provided evidence for an association between LAN and BP. Increased LAN was linked to significantly higher nighttime SBP (120.8 vs. 116.5 mmHg, *P* < 0.01) and DBP (70.1 vs. 67.1 mmHg, *P* < 0.01) compared with the darker group independently of potential confounding factors.^166^

### 4.3 Cardiovascular consequences of light exposure

While the impact of light exposure on the circadian clock, melatonin production and alertness are well-studied, several laboratory studies have examined the effects of light exposure on cardiovascular parameters,^152^ indicating that light can directly or indirectly influence heart rate,^167,168^ heart rate variability (HRV),^152,168–170^ and BP.^171,172^ It has also been shown that effort-related cardiovascular responses, as assessed by cardiac impedance during daytime light exposure, as well as mental task ability, can be influenced by light.^173,174^ A recent study found that overnight light exposure during sleep (at 100 lux) can increase heart rate, decrease HRV, and increase insulin resistance the next day.^168^

### 4.4 A question of timing, dose, and light parameters

With several laboratory studies indicating an acute influence of light exposure, it is worth pointing out that these studies differ in their exact protocol, light exposure parameters (and their reporting^175^) analysis procedures, and outcomes. Consequently, while meta-analytic efforts summarizing the effects of light on circadian phase shifting, melatonin production and alertness have produced convergent results,^176^ no such analysis exists for cardiovascular studies. Moreover, a key challenge lies in converting the well-defined light exposure levels set in laboratory studies to actual exposures in the field. While in laboratory studies, light exposure can be very tightly controlled,^177,178^ the actual light exposure of people can vary significantly due to numerous confounding factors.^179,180^

This poses a difficult challenge for estimating the actual light exposure an individual receives over 24 h, as environmental measurements can at best approximate the mean light exposure an individual has received. As a consequence, wearable light loggers have been developed to capture personal light exposure,^181^ and this is an active area of research.^182^

### 4.5 Mitigation strategies

The evidence base for the effects of light exposure on cardiovascular health is methodologically somewhat heterogeneous, and the exact exposure parameters are non-trivial to estimate unless personal light exposure measurements are performed. Nevertheless, nighttime light exposure should be avoided, given its known disruptive effects on physiology and the evidence from several large-scale epidemiological and field studies.^144–153^ The recommendations of expert consensus can be summarized by the motto ‘bright days and dark nights’, which means for the daytime minimum melanopic EDI (Equivalent Diffuse Illuminance) of 250 Lux, for evening 10 Lux, and for nighttime 1 Lux.^176,183,184^ Spitschan et al. developed a series of consensus statements on light exposure and its impact of health endorsed by several leading organizations, which can be taken up by public health multipliers organizations, which can be taken up by public health multipliers.^184^

In particular, the ‘dark nights’ aspect can be realized through several different interventions. At the legislation and regulation level, it is clear that better outdoor illumination is needed to minimize the presence of unnecessary outdoor illumination in the urban environment.^185^ Precisely, this should translate into different design practices, as summarized by a joint set of recommendations from the International Dark-Sky Association and the Illuminating Engineering Society, stating that light should be helpful to (use light if it is needed), targeted (direct light where it is required), low level (light no brighter than necessary), controlled (use light when it is needed) and have the right colour (use warmer lights where possible).^186^ Short-term personal solutions involve the use of window blinds and thick curtains to block light entering the bedroom, as well as the use of sleep masks.

In summary, nighttime light exposure can negatively affect cardiovascular health, with converging evidence from laboratory, field, and epidemiological studies. Hypertension specifically has been associated with nighttime light exposure.

The following steps are recommended to advance translational research:

- Standard protocols and reporting schemas should be developed to ensure common reporting of the cardiovascular effects of light in laboratory, field, and epidemiological studies.
- Limitations of using satellite imagery for predicting personal light exposure should be explored through careful measurements and/or simulations.

To translate the research into public health, the following steps are recommended:

- Light exposure at the wrong time needs to be recognized as a key disruptor of physiology, with interventions at many levels being possible
- Public health messages should focus on education and awareness.^184,187^

## 5. Toxic metals

**Figure 4:**
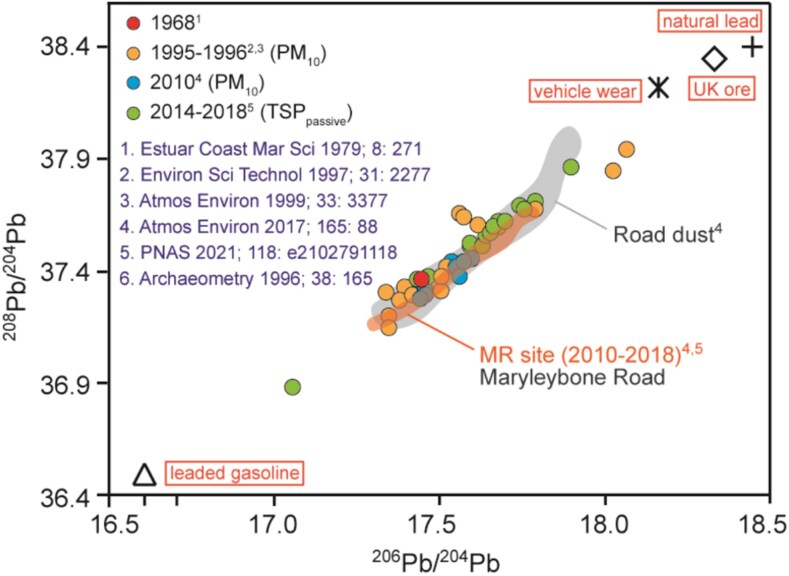
Contribution of 20th century lead deposition to atmospheric environment in London. Marleybone Road is a thoroughfare with very heavy traffic in central London within the City of Westminster. The isotopic ratios of lead in dust particles allow to identify the origin of the lead contamination. Reproduced with permission from reference.^189^

Lead is the prime contaminant among all toxic metals threatening human health, because globally its presence is ubiquitous in the working or living environment. Historically, lead was released in pyrolytic zinc ore refinement and was used as constituent of indoor and outdoor paints, additive to fuels, in conduits distributing drinking water, and as corrosive-resistant building material. The production of ammunition and lead-acid batteries continues to rely heavily on lead. In developed countries, drafting and reinforcing regulations to prohibit lead took until the end of the previous century, while legislation or its enforcement lag behind in middle- and low-income countries.^188^ The major source of exposure in western countries today is lead-containing coarse and fine particulate matter that has accumulated over the past centuries (*Figure 4*). In contrast, in other countries, the gastrointestinal route is an exposure vector.^189,190^

Cadmium is associated with hypertension in some large observational datasets (including NHANES), whereas the long-running Belgian CadmiBel findings have been interpreted as inconsistent with a clear blood-pressure effect—highlighting ongoing uncertainty across populations and study designs.^191,192^ Nevertheless, cadmium causes renal dysfunction (aetiology of secondary hypertension) and by the changes in renal function leads to osteoporosis and is a cancerogenic.^193–196^ Comparable heterogeneity has been reported for arsenic, where systematic reviews/meta-analyses suggest an association with hypertension prevalence but note inconclusive findings for continuous BP outcomes in parts of the literature.^197,198^ Deficiency of selenium might be an underestimated risk factor for the development of high BP in European men.^199^ Evidence regarding copper is likewise contradictory, with NHANES-based analyses reporting largely null or non-robust associations after accounting for key covariates (including antihypertensive treatment), while other approaches do not uniformly support a hypertensive effect.^200^

The most extensively studied source of environmental pollution with a long-standing research history is lead exposure and its association with BP in humans. This section focuses on the association of hypertension and its complication with lead exposure in adults.

### 5.1 Epidemiological evidence

#### 5.1.1 NHANES

The National Health and Nutrition Examination Survey (NHANES) III data from 1988 to 1994^201^ indicates how metrics covering the adverse health effects of lead exposure in general population depend on assumptions.^202^ The hazard ratio (HR) for mortality over a 19.3-year follow-up period, associated with a baseline blood lead increment (10th–90th percentile: 1.0–6.7 μg/dL), was 1.37 (95% CI 1.17–1.60).The population-attributable risk fraction (PAF) related to BL was 18.0% and the number of attributable deaths (NAD) was 412 000 per year. However, because the association sizes were computed and completely ignored the drastic fall in BL among US adults, this NHANES III analysis inflated estimates by the range.^201^ Surprisingly, given the pathophysiology of lead-related hypertension, these findings were not affected by adjustment for BP or hypertension.^201^ The analysis of the 2-year NHANES examination cycles (1999–2020), which included 34 806 individuals, accounted for the sharp BL decrease and included a mediation analysis in consideration of the direct pathway associating BL with mortality and the indirect pathways running via BP or socioeconomic status (SES).^203^ In time-stratified analyses, even though BL decreased from 1.76 µg/dL (1999–2004) to 0.93 µg/dL (2017–2020), the proportion of general population with BL <1 µg/dL increased from 19.2% to 63.0%. Total mortality was not associated with BL, while cardiovascular mortality was related to BL in the 1999–2000 cycle (HR 1.44 [95% CI 1.01–2.07]), but not thereafter. BL was directly related to cardiovascular mortality, while the indirect BL pathway via BP was insignificant. Low SES was directly associated with BL and cardiovascular mortality, but the indirect SES pathway via BP did not reach significance in 2007–2010. Moreover, from 1999–2004 to 2017–2020, BL-related cardiovascular PAF decreased from 7.8% (0.17–14.4%) to 2.5% (0.05–4.7%) and NAD from 53 878 to 7539 deaths.^203^

#### 5.1.2 GBD reports

Global burden disease (GBD) study 1999–2010 proposed that for the individuals aged 25 years or older, there is a causal association between SBP and lead exposure with 0.67 million deaths (UI: 0.58–0.78 million) and 0.56% of DALYs lost (UI: 0.47–0.66%) attributable to environmental lead exposure.^204^ The GBD investigators, however, indicated some possible limitations of their results.^204^ Moreover, the issue of residual confounding requires calculating PAF for risk factors groups, instead of for a single risk indicator. In fact, cardiovascular risk factors and various exposures (i.e. environmental pollutants) cluster within individuals. Then, GBD^205^ or GBD-associated^206,207^ publications redirected the topic of lead-associated health disorders to ischaemic heart disease. The suggested pre-industrial BL concentration in man was probably as low as 0.016 µg/dL. These conclusions were based on extrapolation by linear regression of BL on bone lead in data obtained from humans exposed to lead environmentally or occupationally. However, stroke, the major complication closely related to BL level was not considered as primary endpoint.

#### 5.1.3 Prospective study in workers

The Study for Promotion of Health in Recycling Lead (SPHERL) was the first prospective study which demonstrated the health effects related to occupational lead exposure in 234 newly hired workers in battery-recycling plants without known previous exposure.^208^ In 2 years follow-up BL increased by 3.2-times from the baseline concentration of 4.35 μg/dL. This BL increment, however, was not associated with increase of BP or hypertension incidence, and renal function worsening. It should be emphasized that SPHERL study included participants working under Occupational Safety and Health Act (OSHA) regulations who were relatively young (mean age 28 years) and predominantly men (91%). In meta-analysis included 58 518 subjects recruited from the general population chronic exposure doubling of BL was associated with a marginally higher BP, on average 1.0 mmHg SBP (95% CI 0.5–1.4 mmHg) and 0.6 mmHg DBP (0.4–0.8 mmHg).^209^ In NHANES VI, BL doubling was associated with higher SBP/DBP (+0.76/+0.43 mmHg [95% CI 0.38–1.13]/[0.18–0.68])^210^ (more data available in Supplementary material online, *Table S1*).

### 5.2 Pathophysiology

#### 5.2.1 Lead toxicokinetics in humans

General population exposure to lead through inhalation and ingestion depends on urbanization, dietary habits and socio-economic position.^211^ After reaching the lung alveoli, the finest dust particles easily pass the air–blood barrier and are body-wide distributed via the blood stream. Occupational exposure is mainly due to coarse aerosols that deposit in the upper airways and then translocate to the gastro-intestinal tract by mucociliary clearance (5–10% uptake).

There is very long-half life time of lead in a human body. Approximately, 95% of lead is accumulated in bones, from where it is eliminated over 20–25 years.^212^ Almost 99% of BL is carried by red blood cells, reflecting the recent exposure over the past 1–2 months and the amount of lead released and recirculated from bone deposits. Both bone lead and BL increase with age. Bone lead correlates with BL, explaining around 20% of its variance. The latter reflects the balance between osteogenesis and osteolysis, which depends on numerous endogenous and environmental factors. The significant delay in the change in blood lead concentration in response to a decrease of environmental lead exposure is due to the recirculation of calcium from the bones.

#### 5.2.2 Pathogenic mechanisms

Hypertension and hypertension-related cardiovascular and renal complications are the prime pathophysiological mechanisms of lead exposure. Based on in vivo and in vitro studies the potential mechanisms of chronic lead exposure on hypertension and CVD are proposed: oxidative stress promotion, nitric oxide availability down-regulation, nitric oxide signalling impairment, adrenergic activity augmentation, endothelin production up-regulation, renin-angiotensin system alteration, vasoconstrictor prostaglandins up-regulation, vasodilator prostaglandins down-regulation, inflammation promotion, vascular smooth muscle Ca^2+^ signalling interference, endothelium-dependent vasorelaxation down-regulation, and modification of vascular response to vasoactive agonists.^213^ Estimated increase in SBP/DBP was subtle [1.0/0.6 mmHg (95% CI 0.5–1.4/0.4–0.8 mmHg)] in response to 2-fold increase in BL based on meta-analysis of 31 studies, involving 58 518 participants. All studies pooled in this meta-analysis were published before 2001, when stringent legislation regulating lead exposure started to make an impact.

Environmental lead exposure in American adults was not recognized as a significant risk factor of hypertension development by NHANES 2003–2010 analyses, which showed ethnic inconsistency in the associations between BP and BL due to failing to account for relevant confounding factors.^210^ In the review of five cross-sectional studies SBP (0.26 mmHg; CI 0.02–0.50 mmHg) and risk of hypertension (OR: 1.04; CI 1.01–1.07) were associated with bone lead levels (+10 µg/g).^214^ However, based on the evidence from the same researchers, relation of cardiovascular complications with BL was suggestive at best and insufficient to infer causality.^215^ In a prospective population study among 728 individuals during 5.2 years follow-up, BL decreased by 32% from the baseline level of 8.7 μg/dL (range: 1.7–72.5 μg/dL). BP levels assessed both by auscultatory or 24-h ABPM methods in this study were not related to BL at baseline or at follow-up.^216^

Other potential mechanisms contributing to the association between CVD and lead exposure are speculative and are only extrapolated from experimental studies in cell or animal models. Proposed mechanisms include oxidative stress and inflammation, which can lead to neurotoxicity, endothelial dysfunction, or defective DNA repair.^217,218^ Lead has the ability to either substitute for or compete with essential divalent cations, showing a preference for Ca^2+^,^217,218^ which may explain the small decrease in left ventricular strain and strain rate observed in a population study.^219^ Several single nucleotide polymorphisms, epigenetic modifications and interference with regulatory RNA molecules are also related to individual susceptibility to lead toxicity.^218,220^

### 5.3 Mitigation strategies

The use of lead in paints was banned in 1976, and its use as an additive to gasoline was prohibited by 2000.^221^ Lead was also eliminated from building materials, drinking water pipes, and metal food packaging. The recycling of lead batteries and other lead waste became mandatory. These changes have resulted in a decrease in blood lead levels among American adults from 13.1 µg/dL in NHANES II (1976–1980) to 2.76 µg/dL in NHANES III (1988–1994), to 1.64 µg/dL in NHANES IV (1999–2002), and to 0.93 µg/dL (2017–2020).^203,210^

The US Occupational Safety and Health Administration (OSHA) Standard provided guidance to prevent and decrease lead exposure at the workplace: regular medical monitoring, adequate workplace ventilation, and the obligatory use of personal protective equipment. The European Union Directive (6417/23), which was recently adopted, amends Council Directives 98/24/EC and 2004/37/EC with regard to occupational exposure limits for two important substances: inorganic lead compounds and diisocyanates. The new directive proposes reducing the permissible BL threshold from 70 to 30 µg/dL by 2028, and to 15 µg/dL thereafter, and decreasing the level of lead in air from 0.15 to 0.03 mg/m^3^. For female workers of childbearing age, the BL level limit was set at 4.5 µg/dL to mitigate reprotoxic effects. It is alarming that legislation to protect workers with occupational lead exposure is still under review or is only loosely enforced in middle- and low-income countries.^188^

In summary, lead is a ubiquitous metal worldwide with high toxicity. Lead influences not only the cardiovascular system in adults, but also most other organs. Based on the evidence from observational studies, although since the beginning of the 21st century, massive progress has been made in reducing environmental and occupational lead exposure, further lowering exposure to practicable limits should emphasized in the future regulations. In addition to eliminating the remaining undetected sources of lead exposure, environmental policy should focus on reducing exposure to lead particles accumulated over the past century (see *Figure 4*), as well as educating and empowering disadvantaged social groups, particularly in low- and middle-income countries. The success of mitigation strategies in reducing lead exposure and its adverse health effects provides hope and a positive message about the value of mitigation strategies in the case of other environmental pollutants.

## 6. Climate change

Climate can be described by several major meteorological factors, such as ambient temperature, humidity, and atmospheric pressure. These three factors play a key role in evaluating climate change. Although climate also describes several other meteorological factors, such as rain, snow, etc., temperature, humidity, and atmospheric pressure can be accurately measured. However, studies on the health impact of climate change often involve season as an integrated factor of climate change.^222^

### 6.1 Epidemiological evidence

In seasonal countries and regions, climate change over seasons may have a profound impact on the epidemiology and management of hypertension. With global warming in the past several decades, climate change has become even greater than decades ago.^223^ This situation might be getting worse, if global warming continues in the following years.^224^

#### 6.1.1 Climate change and hypertension prevalence and incidence

##### 6.1.1.1. Temperature

Several previous studies have shown that outdoor temperature is associated with fluctuations in BP. In a cross-sectional study involving 57 375 participants (aged 35–79 years) enrolled in the China Kadoorie Biobank prospective study from the south-east coastal region of China, annual outdoor temperature ranged from −2.9 to 33.7°C, and the overall winter–summer SBP/DBP difference was as much as 15.7/6.8 mmHg.^225^ Each 10°C lower temperature was associated with 14.1% higher prevalence of newly detected hypertension and 13% lower control rate of hypertension (SBP <140 mmHg and DBP <90 mmHg) among patients with previously diagnosed hypertension.^225^ The absolute differences in BP due to indoor or outdoor temperature changes are about 1 mmHg (more data are available in Supplementary material online, *Table S1*).

When ABPM was applied the problem of temperature influence on BP and hypertension becoming even more complex. In the study of 1395 patients living in a mild-climate geographic area in Italy also higher BP were observed during wintertime as compared to summer month.^226^ However, it appears that nighttime BP and the prevalence of isolated nocturnal hypertension was higher in warm than in cold seasons (15.2% vs. 9.8%, *P* = 0.003).^226^ The most probable explanation of these findings is that heat may affect sleep quality, which, in turn, contributes to nighttime BP elevation. Similar results were observed in 1054 untreated Chinese hypertensive patients, in whom the prevalence of isolated nocturnal hypertension was 21.9% in summer and 7.1% in winter (*P* < 0.001).^227^ Several studies investigated the association between the indoor temperature and BP. In 10 242 Yemen participants, each 1°C increase in indoor temperature was associated with a 0.2% reduced hypertension prevalence (age and sex adjusted OR of 0.98; 95% CI 0.96–0.99).^228^ In a country-wide, population-based study involving 2047 participants enrolled in the 2010 Scottish Health Survey, individuals whose households were below 18°C had a higher risk of high BP (OR: 2.08, 95% CI 1.26–3.43, *P* = 0.004) and the odds increased further if the household was even below 16°C (OR: 4.92, 95% CI 1.97–12.24, *P* = 0.001). The population attributable risk of indoor temperature less than 18°C was 9.3%.^229^ The results of the above studies consistently show that BP and prevalence of hypertension are in inverse relationship with ambient temperature.

Anthropogenic global warming is expected to increase population exposure to high ambient temperatures and heatwaves, which has direct implications for BP regulation and hypertension management. Strongly emphasized is the problem of heat waves, which may increase hypertension incidence by 9.7%, influence BP and its regulation especially in vulnerable groups including elderly, young children, and people with chronic cardiovascular, pulmonary, and kidney diseases.^230^ In a recent meta-analysis and systematic review, a 1°C rise in temperature was associated with a 2.1% increase in CVD-related mortality and a 0.5% increase in CVD-related morbidity. Cause specific analyses showed positive associations between high temperatures and cardiovascular disease related mortality across all groups considered, apart from hypertensive diseases.^231^ Meanwhile, in the observational study in elderly individuals from South Korea during the 2018 heatwave, a 1°C increase in indoor temperature was associated with a 0.44 mmHg (95% CI 0.04–0.84 mmHg) lower DBP, though not SBP, in 60 patients with hypertension.^232^

Another manifestation of climate change, i.e. short-term, high-amplitude temperatures swing—often operationalized as diurnal temperature range (DTR) is increasingly recognized as relevant to BP control and hypertension risk. In a study of 46 609 adults, higher DTR was linked to higher SBP and pulse pressure with stronger effects in the hot season.^233^ Study complementing this finding (China Hypertension Survey) showed that SBP and pulse pressure increase due to short term daily temperature variability was more pronounced in prehypertension and hypertension.^234^

##### 6.1.1.2. Seasonal variation

Seasonal variation in BP is a well-recognized global phenomenon, with BP generally higher during colder months and lower during warmer months. In a systematic review and meta-analysis of 47 studies, the pooled summer-winter differences in systolic/diastolic BP (SBP/DBP) indicated that BP was higher in winter by 5.6/3.3 mmHg for clinic measurements, 3.4/2.1 mmHg for daytime ambulatory measurements, and 6.1/3.1 mmHg for home BP measurements, whereas nighttime ambulatory BP was 1.3/0.5 mmHg lower in winter than in summer.^235^ These findings highlight the importance of accounting for seasonal effects in BP monitoring and in the individualized management of hypertension, particularly within the framework of precision medicine.^236–238^

##### 6.1.1.3. Atmospheric pressure and humidity

In addition to outdoor and indoor temperature, several other climate factors, such as atmospheric pressure and humidity, have also been studied for the association with BP. In a Japanese study, 165 participants underwent ABPM for 7 days. The BP values and circadian BP variables were not different between the lowest (1013.6 ± 0.4 hPa) and highest days of atmospheric pressure (1023.6 ± 0.3 hPa).^239^ Similarly, there was no association between BP variability and barometric pressure in the study of Jehn and coworkers.^240^ However, Weinbacher et al.^241^ demonstrated a negative correlation between atmospheric pressure and BP.

It is suggested that air humidity may influence cardiovascular health. Firstly, the effectiveness of metabolic heat elimination processes from the human body is slowed down by high humidity.^242^ Moreover, humidity may also modulate air pollution health effects.^243^ On the other hand, there is a lack of clear confirmation for the association between changes in air humidity and CVD.^244–247^

### 6.2 Pathophysiology

BP increase and hypertension development related to climate change are mediated by pathomechanisms including SNS reaction to temperature change and activation of RAAS by lower temperatures.^248^ According to a recent review, three fundamental neurohormonal regulators of BP, namely the SNS, the RAAS, and the vascular endothelial system (the endothelin and nitric oxide pathways), play role in the pathogenesis of hypertension in relation to seasonal variation.^249^ Cold exposure activates the SNS, leading to increased catecholamine release and cutaneous and systemic vasoconstriction, which reduces heat loss but increases total peripheral resistance and, consequently, BP. SNS activation also stimulates the RAAS via β-adrenergic signalling in the juxtaglomerular apparatus, further promoting vasoconstriction and sodium retention. In parallel, cold exposure impairs endothelial function by reducing endothelial nitric oxide synthase activity and nitric oxide bioavailability, while increasing endothelin-1, a potent vasoconstrictor.^250^

The increase of plasma norepinephrine due to acute cold exposure confirmed the SNS involvement in BP response on low temperature.^251^ The role of RAAS in the increase in acute cold-induced BP remains controversial. Some, but not all, studies have shown that renin, angiotensin II (Ang-II), and/or aldosterone levels increase under acute cold exposure. Nonetheless, both the SNS and RAAS are steadily activated during the long-term cold exposure. The increased NE, Ang-II and aldosterone are driving factors for the vascular constriction and arterial hypertension.^237,249^ Inflammatory pathways may additionally contribute, as cold exposure has been shown to increase interleukin-6 expression, which is implicated in cold-induced hypertension and associated cardiac and renal injury.^252^

The protective of heat waves to hypertensive mortality can mainly be attributable to the leading mechanism of heat action in the cardiovascular system, which is a fall in BP due to vasorelaxation and dehydration. Nonetheless, the potential protective role of hypertension for heat-related cardiovascular problems should be interpreted with caution as patients with hypertension commonly have other CVDs. In a 15-year time-stratified case-crossover study, He et al. reported that nighttime heat exposure was associated with an increased stroke risk.^253^ The elevated blood viscosity during hot night might increase the risk of ischaemic strokes by promoting blood clot formation, reducing blood flow efficiency, and contributing to hypertension and atherosclerosis.^254^

### 6.3 Mitigation strategies

In light of the previously cited data on the higher prevalence of hypertension in cold seasons, it is important to note that there is a stronger association between indoor than outdoor temperature and BP in cold seasons.^229,255,256^ The 2018 WHO housing and health guidelines emphasized the critical role of managing indoor housing temperature to protect from harmful health effects of cold.^257^ The WHO recommends the optimum indoor temperature for countries with temperate or colder climates as more than 18°C.^257^ In addition, the WHO conditionally recommends installing efficient and safe thermal insulation in new housing and retrofitting it in old housing in climate zones with a cold season. However, thermal insulation might need to be more strongly acknowledged as a means to control BP levels and variability, because of the dual role in both raising and stabilizing indoor temperatures.^258^ In an intervention study in Japan, Umishio et al.^259^ found that insulation retrofitting (indoor temperature in the morning rise by 1.4°C) significantly reduced morning home SBP/DBP by 3.1/2.1 mmHg and evening home SBP/DBP by 1.8/1.5 mmHg. The indoor but not outdoor temperature instability was significantly associated with the diurnal and day-by-day BP variabilities.^260^ In other study among elderly Japanese, instruction for home heating till 24°C before estimating rising decreased significantly SBP/DBP by 4.43/2.33 mmHg.^261^ Thus, instructing the elderly in the use of heaters could effectively reduce BP. The combination of a high insulation level and continuous heating use is potentially important to reduce indoor temperature instability and therefore BP, and should be recommended.

On the other hand, there is the problem of high temperatures, especially heat waves, which are often associated with hypertension. Heatwaves are considered as dangerous for health especially vulnerable groups including elderly, young children and people with chronic cardiovascular, pulmonary and kidney disorders.^230^ In fact, according to a meta-analysis and systematic review recently published in *LANCET*, a 1°C rise in temperature was associated with a 2.1% increase in CVD-related mortality and a 0.5% increase in CVD-related morbidity. However, this does not apply to hypertension, for which no increase in mortality was found, and the authors even indicated a protective role for hypertension concerning heat-related cardiovascular morbidity.^231^ This is mainly due to the leading mechanism of heat action in the cardiovascular system, which is a fall in BP due to vasorelaxation and dehydration. The potential protective role of hypertension for heat related cardiovascular problems should be interpreted with caution as patients with hypertension commonly have other CVDs. The widely used guidelines for heat exposure protection should also be implemented for hypertensive individuals. Moreover, the treatment with BP lowering drugs should be considered. According to ESH 2023 Guidelines BP values in the summer do not pose specific problems to the patient but may require a supplementary medical visit. If lower BP values are associated with dizziness or fatigue, patients may be advised to modestly down-titrate drug treatment, especially diuretics.^22^

The problem with BP lowering treatment and seasonal temperature variations is however more complex.^235,262–264^ Previous studies showed that there was indeed seasonality in the antihypertensive treatment effect.^265,266^ A recent seasonality ESH Consensus recommends treatment down-titration when BP is below the recommended target in hot seasons. However, substantial BP drop should be confirmed by repeated office measurements, and preferably with home or ABPM. Other reasons for BP change must be excluded.^236^ So far, there is no robust evidence in support of specific criteria for the down-titration of antihypertensive medications.

The consensus of the European Society of Hypertension (ESH) working group advocates that tapering of antihypertensive medications should be carefully considered in patients with SBP <110 mmHg (office, home, or ambulatory).^236,237^

In light of the previously discussed data, ABPM and home blood pressure (HBP) monitoring should be recommended for evaluating BP changes due to climate change. This also applies to ABPM use for detecting isolated nocturnal hypertension and HBP for the early identification of hypertension due to temperature change.^138,237,267–269^

Regular BP monitoring and timely medication adjustments are key to mitigating seasonal effects. In the HOMED-BP study, Hanazawa et al. showed that earlier titration or tapering of antihypertensive therapy reduced the summer-winter BP difference and improved cardiovascular outcomes.^270^ As winter approaches, up-titrating medication may help prevent seasonal BP increases.^271^ Similarly, in a 12-week study of irbesartan/hydrochlorothiazide, Ye et al. reported greater BP reduction when treatment was initiated in autumn or winter than in spring or summer.^266^

In a large-scale study that included over 110 000 volunteer participants, significant seasonal variations in HBP collected using a web-based healthcare platform were confirmed.^272^ Kario and colleagues have also demonstrated that an information and communications technology used for HBP monitoring was effective in the evaluation of seasonal BP changes.^273–275^ This technology helped patients achieve tight BP control and suppressed excessive seasonal BP fluctuations.^273^ Novel digital technologies will probably be the future of hypertension management, including BP fluctuations related to climate change.

**Figure 5:**
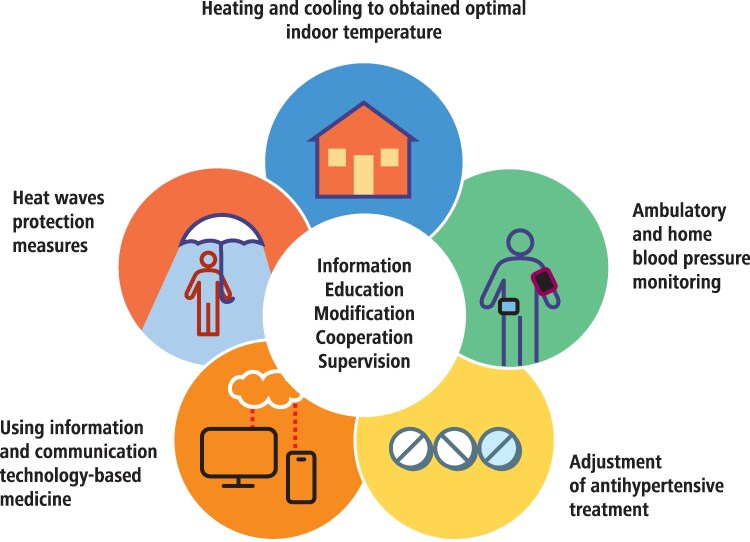
Climate change—summary and recommendations for healthcare providers.

In summary, here is some evidence suggesting effects of climate factors on BP as well as prevalence and control of hypertension. However, the evidence is generally weak, probably because of no or low awareness and because of difficulties in conducting such studies. Future research is required to address this issue with the increasingly used novel technologies for BP monitoring (*Figure 5*).

## 7. Other pollutants

Various chemical compounds have been proposed as potential contributors to hypertension, although evidence from human studies remains limited. For example, a recent analysis of NHANES data demonstrated a significant positive association between urinary biomarkers of non-persistent pesticides—particularly para-nitrophenol, reflecting exposure mainly through residues in fruits and vegetables rather than drinking water—and hypertension in adults. Moreover, combined exposure to multiple pesticide metabolites also linked to higher odds of hypertension.^276^

Similarly, persistent organic pollutants such as dioxins and dioxin-like compounds have been linked to hypertension in NHANES-based observational analyses. In humans, exposure to dioxins occurs predominantly through dietary intake, particularly via animal-derived foods rich in fat, while additional sources include industrial emissions, contaminated soil and dust, tobacco smoke, and occupational exposure. Nevertheless, causal inference is constrained by residual confounding and co-exposure to complex chemical mixtures.^277^

For microplastics, mechanistic and animal data are growing, but robust human studies directly addressing BP are not available.^278^

## 8. Conclusion and perspectives

The need to develop a position paper on the role of environmental pollution—a key component of the external exposome—in hypertension is a result of dynamic development of knowledge in this field, as evidenced by the growing number of epidemiological and experimental studies and publications on this topic. Environmental factors such as air pollution, noise and light pollution, exposure to heavy metals, and climate change have been shown to cause many diseases and multimorbidity. Hypertension occupies a special position in this regard, as it is a functional disorder and a form of dysregulation of systemic haemostasis that occurs earliest before establishing CVD.

For the same reasons, hypertension as a prodromal syndrome of the adverse cardiovascular effects of environmental pollution provides an opportunity for early intervention to prevent the development of complications. In each chapter of this position paper, the epidemiological data have been highlighted to provide comprehensive evidence of the causal—aetiological role of environmental pollution in the development of hypertension. Such a role was first recognized by both the 2023 ESH and the 2024 ESC guidelines. It is crucial to disseminate this way of thinking about environmental pollution among physicians and health professionals. Many basic science studies have provided a pathophysiological explanation for the influence of environmental factors on the development of hypertension and related target organ damage.

From a practical point of view, the most important part of this document is identifying opportunities to mitigate environmental factors’ impact on hypertension and cardiovascular morbidity. Legislative and technological action in this area has for many years been aimed at reducing emissions and exposure to pollutants. In many respects, e.g. the reduction of environmental exposure to lead, the reduction of air pollution rates and associated mortality in the European Union, these actions have been very successful, and the proposed further solutions offer good prospects. Medical action to reduce the adverse effects of environmental pollution on the development of hypertension and other diseases consists primarily of counselling patients to make them aware of the role of environmental factors in disease, how to avoid/reduce exposure to them, the use of individual exposure monitoring and protective measures.

Evidence on the influence of environmental factors on hypertension and the effectiveness of mitigation strategies mainly comes from large-scale epidemiological studies rather than interventional trials. While this enables relatively straightforward population-level recommendations to be formulated to reduce emissions and exposure, translating these findings into personalized guidance remains challenging due to heterogeneity in patient characteristics and complex, multi-pollutant exposure profiles. Nevertheless, recommendations at the individual level are necessary to raise awareness of the environmental determinants of hypertension and emphasize the potential of individual actions to reduce exposure and contribute to overall emission reductions through behavioural change.

Physician-led activities in this area include implementation of scientific society guidelines for strict control of hypertension risk factors in exposed populations, emphasis on more frequent screening of BP levels, and use of modern BP measurement methods for early detection of hypertension in populations with known exposures. The latter requires the involvement of both health professionals and patients in actively seeking information on environmental exposure levels available from monitoring systems (currently, air and noise pollution data). Environmental exposure assessment should be recommended for all patients already diagnosed with hypertension, as this is a particularly vulnerable group.

In addition to controlling conventional risk factors, reducing exposure to environmental risk factors (discussed in detail in the chapters on different aspects of environmental pollution) should be particularly recommended in this group. In light of the available literature, groups that are also particularly sensitive to environmental exposures (the elderly, pregnant women, children, shift workers or people working outdoors) need greater medical attention with regard to both hypertension and concomitant diseases.

Finally, future updates of this document may include even wider understanding of environment including relations with others, be it at home, at work or in the context of war, chronic violence and migrations affecting entire populations. This is particularly relevant in view of the growing number of patients referred for HTN in the context of burnout, harassment at work or at home, as well as migrants and refugees with resistant, difficult-to-treat or malignant HTN. These different situations can lead to the development of post-traumatic stress disorder (PTSD), which in turn is associated with an increased risk of HTN and CVDs.^279–282^ Complex interactions between exposure to man-made trauma with or without PTSD, pollution, noise and climate change and their joint impact on human health and well-being deserve further investigation, both at individual and societal level, in order to better orient public health policies in a globalized society.

With its limitations, we believe that this already comprehensive document will be useful in daily practice and self-education and will contribute to better patient management.

## Supplementary Material

cvag061_Supplementary_Data

## Data Availability

No new data were generated or analysed in this study.
