# Supplementary material for: The impact of environmental pollution and climate change on hypertension: a position paper by the European Society of Hypertension (ESH) Working Group on Environment in Hypertension
Source: Cardiovasc Res. 2026 Mar 20;122(7):835–57. doi: 10.1093/cvr/cvag061 (PMC13196876; doi:10.1093/cvr/cvag061)
Supplement: cvag061_Supplementary_Data [file cvag061_supplementary_data.docx]

**Supplementary Table 1**

**Estimated blood pressure changes as a consequence of environmental pollution exposure**

| **Environmental factor** | **Best available scientific quantitative estimate of BP change** | **Evidence source** |
| --- | --- | --- |
| **Air pollution** | **+1.39 mmHg SBP** and **+0.90 mmHg DBP** per **10 µg/m³ PM₂.₅** increase (meta-analysis)  + 2.54 mmHg SBP and + 1.36 mmHg DBP per each IQR μg/m^3^ **PM₂.₅ increase**  + 2.38 mmHg SBP and + 1.56 mmHg DBP per IQR μg/m^3^ SO_4_^-2^ increase  + 1.39 mmHg SBP and + 0.83 mmHg DBP per IQR μg/m^3^ NO_3_^–^ increase  + 2.17 mmHg SBP and + 1.26 mmHg DBP per IQR μg/m^3^ NH_4_^+^ increase  + 3.76 mmHg SBP and + 1.64 mmHg DBP per IQR μg/m^3^ OM increase  + 3.87 mmHg SBP and + 2.11 mmHg DBP per IQR μg/m^3^ BC increase | Liang R, *et al*. J Hypertension 2014; 32: 2130-2140  Fu L, *et al*. Environ Int 2024; 184:108464 |
| **Noise pollution** | **Road traffic noise**  +1.06 mmHg SBP and + 0.40 mmHg DBP for every 10 dB increase in Lden  +3.03 mmHg SBP and + 0.68 mmHg DBP for every 10 dB increase of Ln  **Aircraft noise**  +1.93 mmHg SBP and + 1.08 mmHg DBP for every 10 dB increase Lden | Kupcikova Z, et al. Eur Heart J 2021;42:2072-2084.  Li S, et al. BMC Public Health 2021;21:815.  Kourieh A, et al. Occup Environ Med 2022; 79:268-276. |
| **Light pollution** | +0.59 mmHg SBP and +0.85 mmHg DBP per each IQR of LAN increase  +0.52 mmHg SBP and + 0.58 mmHg DBP per each IQR increase for ALAN | Wang R, et al, J Clin Hypertens 2024;26:134-144  Palomar-Cros A, et al. Am J Epidemiol 2024;194:963-974. |
| **Toxic metals** | **Lead**  +1.0 mmHg SBP and + 1.6 mmHg DBP at doubling BL concentration  +0.76 mmHg SBP and +0.43 mmHg DBP at doubling BL concentration  **Arsenic**  **+4.03 mmHg SBP and +1.52 mmHg DBP at high vs low** chronic arsenic exposure  **Cadmium**  +2.17 mmHg SBP and +1.46 mmHg DBP for doubling of blood cadmium concentration | Nawrot TS, et al. J Hum Hypertens 2022;16:123-131.  Hara A, et al. Hypertension 2015;65:62-69.  Zhao J, et al. Environ Pollut 2021;289:117914.  An HC, et al. Ann Occup Environ Med 2017;29:47. |
| **Temperature / Seasonal variation** | **Outdoor/indoor temperature**  **- 0.31 mmHg SBP per 1°C** increase of indoor temperature  **- 0.19 mmHg SBP per 1°C** increase of outdoor temperature  -0.37 mmHg SBP and -0.22 mmHg DBP per 1°C increase in indoor temperature  -033 mmHg SBP and -0.12 mmHg DBP per 1°C increase in indoor temperature over outdoor temperature  -3.1 mmHg SBP and -2.1 mmHg DBP per 1°C increase in indoor temperature due to insulation and retrofitting despite 0.2°C decrease of outdoor temperature  -0.44 mmHg DBP per 1°C increase in indoor temperature in elderly with hypertension  -4.43 mmHg SBP and -2.33 mmHg DBP per 2.09°C increase in indoor temperature due to heating  -1.75 mmHg SBP and -2.05 mmHg DBP per 1°C increase in indoor temperature  -0.20 mmHg SBP and -0.35 mmHg DBP per 1°C increase in outdoor temperature  +0.26 mmHg SBP and +0.13 mmHg DBP per 1°C reduction in ambient outdoor temperature  **Diurnal temperature range (DTR)**  With a 1 °C increase of DTR, SBP and PP increased 0.058 mmHg and 0.114 mmHg respectively, and DBP decreased 0.039 mmHg  With a 2.28 °C IQR increase of DTR +0.31 mmHg SBP and + 0.59 mmHg PP among prehypertensive participants  **Seasonal variations**  +9 mmHg SBP higher during winter than in summer (+6.2 mmHg SBP at each temperature decrease by 10 °C starting from < 5°C)  The pooled summer minus winter SBP/DBP difference was –5.6/–3.3 mmHg in office, –3.4/–2.1 mmHg in daytime ambulatory BP, 1.3/0.5 mmHg in night-time ambulatory BP, and –6.1/–3.1 mmHg in home BP. | Barnett AG, et al, Blood Press Monit 2007; 12:195-203  Kinuta M, et al. Hypertens Res 2023;46:200-207.  Umishio W, et al. J Hypertens 2020;38:2510-2518.  Kim S, et al. Environ Anal Health Toxicol 2020;35:e2020024-0.  Saeki K, et al. J Hypertens 2015;33:2338-2343.  Kim YM, et al. Environ Health Toxicol 2012;27:e2012013.  Wang Q, et al. Sci Total Environ 2017;575:276-286.  Zheng S, et al. Sci Total Environ 2020;730:138987.  Yan X, et al. Environ Int 2024;184:108463.  Yang L, et al. Eur Heart J 2015;36:1178-1185.  Kollias A, et al. J Hypertens 2020;38:791-798. |

Abbreviations: BP, blood pressure; SBP, systolic blood pressure; DBP: diastolic blood pressure; BL, blood lead; 95% CI: 95% confidence interval; SPHERL: Study for Promotion of Health in Recycling Lead; NHANES: National Health and Nutrition Examination Survey, IQR: interquartile range; SO_4_^-2^: sulfate, NO_3_^-^: nitrate, NH_4_^+^: ammonium, OM: organic matter, BC: black carbon; Lden: average noise level during day, evening and night; Ln - average noise level during night; LAN: outdoor light at night; ALAN: outdoor artificial light-at-night; DTR: diurnal temperature range; PP: pulse pressure.
